# Anti-inflammatory effect of Angiotensin 1-7 in white adipose tissue

**DOI:** 10.1080/21623945.2024.2449027

**Published:** 2025-01-13

**Authors:** Nozomi Nishida, Satoru Sugimoto, Satoshi Miyagaki, Chiharu Cho, Madoka Konishi, Takeshi Goda, Mihoko Yamaguchi, Yasuhiro Kawabe, Hidechika Morimoto, Joji Kusuyama, Takuro Okamura, Masahide Hamaguchi, Jun Mori, Hisakazu Nakajima, Michiaki Fukui, Tomoko Iehara

**Affiliations:** a Department of Pediatrics, Graduate School of Medical Science, Kyoto Prefectural University of Medicine, Kyoto, Japan; b Department of Biosignals and Inheritance, Graduate School of Medical and Dental Sciences, Institute of Science Tokyo, Tokyo, Japan; c Department of Endocrinology and Metabolism, Graduate School of Medical Science, Kyoto Prefectural University of Medicine, Kyoto, Japan; d Division of Pediatric Endocrinology, Metabolism and Nephrology, Children’s Medical Center, Osaka City General Hospital, Osaka, Japan

**Keywords:** Angiotensin 1-7, obesity, anti-inflammatory effect, MCP-1, TNF-α

## Abstract

Obesity is a global health concern that promotes chronic low-grade inflammation, leading to insulin resistance, a key factor in many metabolic diseases. Angiotensin 1–7 (Ang 1–7), a component of the renin-angiotensin system (RAS), exhibits anti-inflammatory effects in obesity and related disorders, though its mechanisms remain unclear. In this study, we examined the effect of Ang 1–7 on inflammation of white adipose tissue (WAT) in dietary-induced obese mice. Monocyte chemoattractant protein-1 (MCP-1) produced by white adipocytes and tumour necrosis factor-α (TNF-α) produced by macrophages are pro-inflammatory cytokines and interact to form a pathogenic loop to exacerbate obesity-induced inflammation. We found that Ang 1–7 reduced MCP-1 and TNF-α gene expressions and the number of crown-like structures, which are histological hallmarks of the pro-inflammatory process, in visceral epididymal WAT (eWAT) and reduced circulating MCP-1 and TNF-α levels, accompanied by improvement in insulin resistance, in dietary-induced obese mice. Furthermore, Ang 1–7 reduced MCP-1 and TNF-α secretions in 3T3-L1 white adipocytes and RAW 264.7 macrophages, respectively, which are *in vitro* experimental models mimicking obesity condition. Our results suggest that Ang 1–7 directly acts on WAT to mitigate obesity-induced inflammation. Thus, this study provides novel insights into the underlying mechanism of anti-obesity effects of Ang 1–7.

## 1. Introduction

Obesity has become a global health concern owing to its capacity to result in low-grade chronic inflammation through the infiltration of immune cells such as macrophages, lymphocytes, and neutrophils in insulin-sensitive tissues [1]. Obesity-induced inflammation results in insulin resistance, which is a hallmark of type 2 diabetes, cardiovascular disease, stroke, and cancer [2]. Since visceral white adipose tissue (WAT) is a key source of pro-inflammatory molecules that contribute to systemic insulin resistance, targeting visceral WAT inflammation is a potential approach to treat obesity and related metabolic disorders [3].

The renin-angiotensin system (RAS) is classically recognized for its role in regulating blood volume, electrolyte balance, and systemic vascular resistance [4]. Additionally, the RAS has been shown to play an important role in obesity and insulin resistance. Enhancement of the Angiotensin II (Ang II)-Ang II receptor type 1 (AT1) axis is critically responsible for physiological actions of the RAS and is known to exacerbate insulin resistance [4]. Ang 1–7, an endogenous ligand of Mas receptor, is known to counterbalance the deleterious effects of Ang II signalling and has been reported to exert beneficial effects in obesity-related metabolic disorders [5]. Additionally, the Ang 1–7/Mas receptor axis has been demonstrated to mitigate inflammation in various organs [6]. For instance, Mori et al. demonstrated that Ang1–7 treatment offers protective benefits in diabetic nephropathy by alleviating oxidative stress, inflammation, and fibrosis in *db/db* mice [7]. Feltenberger et al. demonstrated that Ang 1–7 administration enhances metabolic function and reduces inflammation markers in the livers of high-fat diet (HFD)-fed obese mice [8]. A few studies have explored the anti-inflammatory effects of Ang 1–7 on WAT; however, the experimental conditions and findings of these studies are not consistent [9–11]. Furthermore, the underlying mechanisms are not yet well understood, highlighting the need for further investigation.

In this study, we investigated the potential anti-inflammatory effects of Ang 1–7 in the visceral epididymal WAT (eWAT) of dietary-induced obese mice and in *in vitro* obesity models of 3T3-L1 white adipocytes and RAW264.7 macrophages. This study can be instrumental in supporting the therapeutic potential of Ang 1–7 for obesity and related metabolic disorders.

## 2. Results

### 2.1. Ang 1–7 improves glucose tolerance and insulin resistance in HFD-induced obese mice

**Figure 1.**
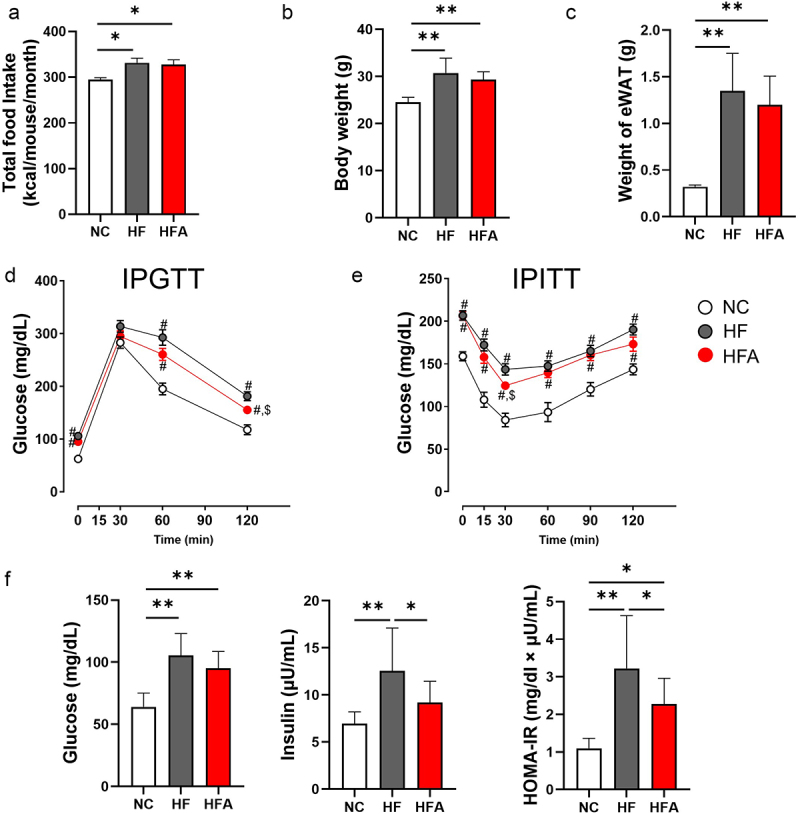
Angiotensin 1–7 (Ang 1–7) improves insulin resistance and glucose tolerance without affecting body weight, food intake, and epididymal white adipose tissue (eWAT) weight in dietary-induced obese mice. (a) Food intake, *n* = 4. (b) Body weight, *n* = 11–16. (c) Weight of eWAT, *n* = 11–15. (d) Levels of blood glucose during intraperitoneal glucose tolerance test (IPGTT), *n* = 10–15. (e) Levels of blood glucose during intraperitoneal insulin tolerance test (IPITT), *n* = 9–15. (f) Levels of blood glucose, serum insulin, and homeostasis model assessment of insulin resistance (HOMA-IR), with sample sizes of *n* = 11–15, *n* = 10–14, and *n* = 10–14, respectively. *p < 0.05, **p < 0.01. ^#^*p* < 0.05 vs. NC. ^$^*p* < 0.05 vs. HF. NC, mice fed a normal chow diet; HF, mice fed a high-fat diet alone; HFA, mice fed a high-fat diet and treated with Ang 1–7.

Ang 1–7 did not affect food intake, body weight, and eWAT weight of HFD-fed mice (Figure 1a–c). Intraperitoneal glucose tolerance test (IPGTT) and intraperitoneal insulin tolerance test (IPITT) were performed to assess glucose and insulin sensitivity, respectively. HFD-fed mice treated with Ang 1–7 (HFA group) exhibited significantly lower blood glucose levels at 120 min in IPGTT and at 30 min in IPITT, as compared to untreated HFD-fed mice (HF group) (*p* < 0.05) (Figure 1d,e). Fasting glucose, fasting insulin, and homoeostasis model assessment of insulin resistance (HOMA-IR), an index of insulin resistance, were significantly increased in HFD-fed mice compared with normal chow diet (NC)-fed mice (*p* < 0.01). Plasma insulin levels and HOMA-IR were significantly lowered by Ang 1–7 treatment in HFD-fed mice (*p* < 0.05) (Figure 1f).

### 2.2. Ang 1–7 reduces eWAT inflammation in HFD-induced obese mice

**Figure 2.**
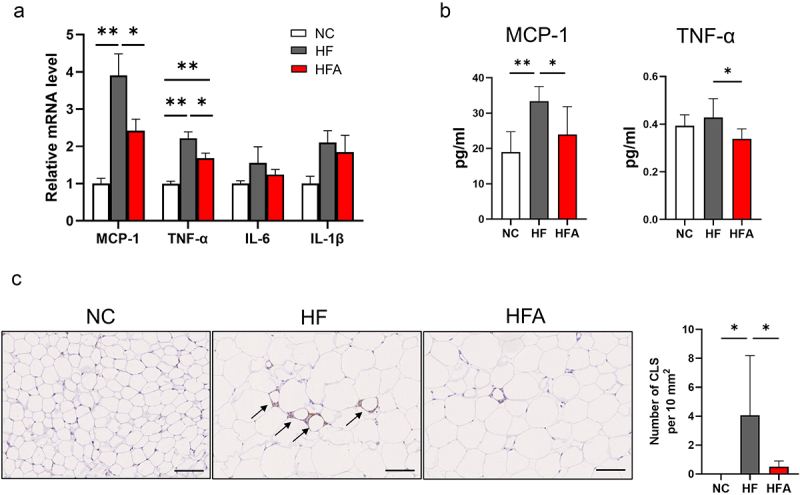
Ang 1–7 reduces inflammation in visceral eWAT in HFD-induced obese mice. (a) Relative mRNA level of MCP-1 (*n* = 6–7), TNF-α (*n* = 6–7), IL6 (*n* = 6–8) and IL1-β (*n* = 6–8) determined using real-time PCR. (b) Serum levels of MCP-1 (*n* = 6–8) and TNF-α (*n* = 6–8). (c) Left panel: representative histology of crown-like structures (CLS, indicated by black arrows) in eWAT of NC, HF,and HFA mice. NC, mice fed a normal chow diet alone; HF, mice fed a high-fat diet alone; HFA, mice fed a high-fat diet and treated with Ang 1–7. Scale bars, 100 μm. Right panel: number of CLS in eWAT (*n* = 5–8). Data are presented as means ± SE. *p < 0.05, **p < 0.01.

We investigated whether Ang 1–7 could mitigate eWAT inflammation in HFD-fed mice. Macrophages play a central role in regulating inflammation in obesity [12]. Monocyte chemoattractant protein-1 (MCP-1), a member of the CC chemokine family, is secreted from adipocytes and plays a major role in recruiting macrophages to adipose tissues, resulting in insulin resistance [13]. Additionally, tumour necrosis factor-α (TNF-α) is secreted from macrophages and impairs insulin signalling, leading to insulin resistance [14]. In this study, HFD feeding significantly increased the gene expression levels of MCP-1 and TNF-α in eWAT (*p* < 0.01). However, the gene expression levels of interleukin-1 beta (IL-1β) and IL-6, both of which are also macrophage-derived pro-inflammatory cytokines [15], were not significantly altered by HFD feeding in eWAT. In the eWAT of HFD-fed mice, Ang 1–7 treatment significantly decreased the gene expression levels of MCP-1 and TNF-α (*p* < 0.05) but did not affect the expression levels of IL-1β or IL-6 (Figure 2a).

The plasma levels of MCP-1 (*p* < 0.05) and TNF-α (*p* < 0.05) were significantly reduced by Ang 1–7 in HFD-fed mice (Figure 2b). Ang 1–7 significantly reduced the number of clown-like structures (CLS), formed by clustering macrophages surrounding a damaged or necrotic adipocyte in eWAT (Figure 2c).

### 2.3. Ang 1–7 decreases MCP-1 secretion in 3T3-L1 white adipocytes through Mas receptor

**Figure 3.**
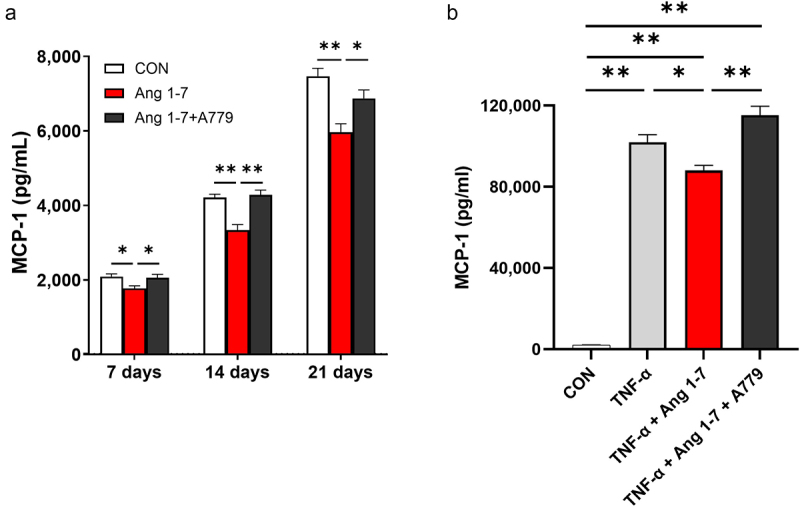
Ang 1–7 reduces MCP-1 secretion in an obese model of 3T3-L1 white adipocytes. (a) MCP-1 concentration in the culture medium of 3T3-L1 adipocytes at 1, 2, and 3 weeks after differentiation, determined by ELISA. CON, control group (vehicle-treated cells), *n* = 7–8; Ang 1–7, cells treated with Ang 1–7 alone, *n* = 7–8; Ang 1–7+A779, cells treated with Ang 1–7 and A779, *n* = 7–8. (b) MCP-1 concentration in the culture medium of TNF-α-treated 3T3-L1 adipocytes, determined by ELISA. CON, control group (vehicle-treated cells), *n* = 7; TNF-α (cells treated with TNF-α), *n* = 6; TNF-α+Ang 1–7, cells treated with TNF-α and Ang 1–7, *n* = 5; TNF-α+Ang 1–7+A779’, cells treated with TNF-α, TNF-α+Ang 1–7+A779, *n* = 5. Data are presented as means ± SE. *p < 0.05, **p < 0.01.

3T3-L1 is a well-established pre-adipocyte cell line and is widely used to study mechanisms of obesity and related pathologies [16]. Lipid accumulation has been reported to gradually increase in 3T3-L1 cells from 1 to 3 weeks following their differentiation. Ito et al. defined 3T3-L1 adipocytes cultured for 1 and 3 weeks after differentiation as non-hypertrophic adipocytes and hypertrophic adipocytes, respectively [16]. We observed a significant reduction in MCP-1 protein levels in the culture medium of 3T3-L1 cells at 1, 2, and 3 weeks post-differentiation upon Ang 1–7 treatment; however, this decrease in MCP-1 level was attenuated by A779 (Figure 3a).

Since TNF-α is known to directly induce insulin resistance in adipocytes [17], TNF-α-treated 3T3-L1 adipocytes have been extensively utilized as an insulin resistance model [18,19]. Thus, we examined the effect of Ang 1–7 on MCP-1 secretion in TNF-α-treated 3T3-L1 adipocytes. Markedly elevated MCP-1 levels in the culture medium were detected in TNF-α-treated 3T3-L1 adipocytes. Ang 1–7 significantly reduced MCP-1 levels in the culture medium of TNF-α-treated 3T3-L1 adipocytes (*p* < 0.05), which was attenuated by A779 (*p* < 0.01) (Figure 3b). Taken together, our results suggest that Ang 1–7 reduces MCP-1 secretion via the Mas receptor in white adipocytes mimicking obese condition.

### 2.4. Ang 1–7 reduces TNF-α secretion in lipotoxic macrophage model

**Figure 4.**
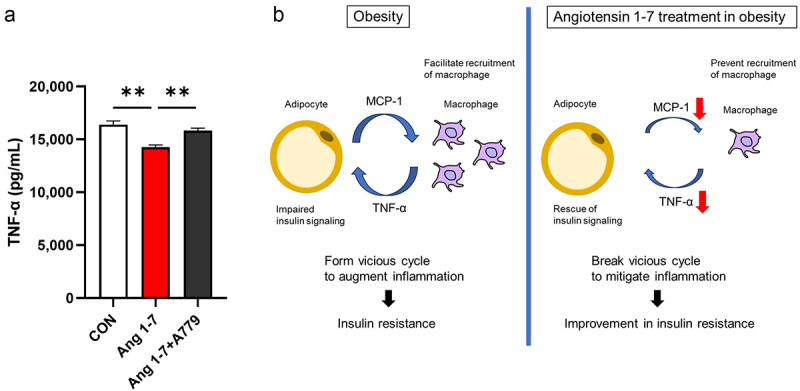
Ang 1–7 reduces TNF-α secretion in a model of macrophage lipotoxicity. (a) TNF-α concentrations in the culture medium of LPS- and PA-treated RAW264.7 cells. CON, cells treated with only LPS and PA (control group); Ang 1–7, cells treated with LPS, PA, and Ang 1–7; Ang 1–7+A779, cells treated with LPS, PA, Ang 1–7, and A779. *n* = 14 for each group. Data are presented as means ± SE. **p < 0.01. (b) Schematic diagram showing the mechanism by which Ang 1–7 reduces inflammation in WAT in obesity. Macrophage-derived TNF-α not only impairs insulin signalling but also stimulates adipocytes to enhance more MCP-1 secretion in obesity. Interaction between adipocyte and macrophage forms a vicious cycle to augment inflammation. Ang 1–7 breaks this vicious cycle by acting on adipocytes and macrophages to reduce MCP-1 and TNF-α secretion, respectively.

Adipose tissue macrophages secrete TNF-α, which plays a crucial role in insulin resistance [14]. Mouse monocyte/macrophage RAW264.7 cells stimulated by lipopolysaccharide (LPS) and palmitic acid (PA) have been used as an established model of macrophage lipotoxicity. Thus, we examined the direct effect of Ang 1–7 on TNF-α secretion in this obesity experimental model. Ang 1–7 reduced TNF-α levels in the culture media of LPS- and PA-treated macrophages, but this reduction was attenuated by A779 (Figure 4a). These results suggest that Ang 1–7 reduces TNF-α secretion via the Mas receptor in macrophages.

## 3. Discussion

Obesity is a pro-inflammatory disease and a major risk factor for various chronic diseases, including type 2 diabetes, non-alcoholic steatohepatitis, cardiovascular disease, and cancer. As obesity progresses, immune cells infiltrate adipose tissue, inducing inflammation and resulting in insulin resistance [20–22]. Ang 1–7, an important component of the RAS, is known to counteract obesity and related metabolic disorders; however, its underlying mechanisms remained unclear [6,7]. Ang 1–7 is known to have a protective role in various inflammatory diseases [7,8,23,24]. Inflammation of visceral WAT is closely associated with systemic insulin resistance in obesity [25]. Few studies have examined the anti-inflammatory effect of Ang 1–7 on visceral WAT; however, the results and experimental model were inconsistent [10,11]. Mori et al. demonstrated that Ang1–7 reduced expressions of MCP-1, TNF-α, IL-1β and IL-6 in visceral WAT of *db/db* mice [10]. Meanwhile, Santos et al. showed that Ang1–7 reduced IL-1β expression and but did not alter TNF-α expression in visceral WAT of HFD-fed transgenic rats expressing an Ang-(1–7)-producing fusion protein [11]. Here, we examined the anti-inflammatory effect of Ang 1–7 on one of the major depots of visceral WAT (i.e. eWAT) in dietary-induced obese mice. We also assessed whether Ang 1–7 directly impacts the *in vitro* obesity models of white adipocytes and macrophages.

In our dietary-induced obese mouse model, HFD feeding significantly increased MCP-1 and TNF-α expression levels in eWAT, whereas IL-1β and IL-6 expression levels exhibited no significant changes. This difference in our results, as compared to those of previous studies, is likely attributable to variations in experimental conditions, including differences in the mouse model and age; specifically, our dietary-induced obese mouse model utilized younger mice compared to those used in previous studies [10,26–28]. MCP-1 and TNF-α play crucial roles in the relatively early stages of inflammation associated with obesity, whereas IL-6 and IL-1β are potentially involved in the later phases of inflammation [29,30]. Thus, we focused our subsequent investigations on MCP-1 and TNF-α, as they appear to be the primary drivers of inflammation in our dietary-induced obese mouse model.

Among the infiltrated immune cells in adipose tissue in obesity, macrophages play a central role in regulating inflammation associated with insulin resistance [12]. MCP-1, a CC chemokine protein, triggers the release of monocytes from the bone marrow and then directs these monocytes towards inflamed tissues where they undergo differentiation into macrophages [31,32]. Overexpression of MCP-1 in mice adipose tissue increases macrophage infiltration in the adipose tissue and exacerbates insulin resistance, whereas MCP-1 homozygous knockout mice exhibit the opposite phenotype [13,33]. In the present study, Ang 1–7 reduced MCP-1 gene expression in eWAT, circulating MCP-1, and the number of CLS in the eWAT of dietary-induced obese mice. Additionally, Ang 1–7 decreased MCP-1 secretion in hypertrophic adipocytes and TNF-α-treated adipocytes, which is a model of insulin resistance. These results suggest that Ang 1–7 directly acts on visceral white adipocytes to prevent infiltration of macrophages in obese mice.

It has been well documented that TNF-α is associated with glucose metabolism and insulin resistance in both animals and humans. TNF-α is mainly secreted from macrophages in WAT and directly impairs insulin signalling [2]. We found that Ang 1–7 reduced TNF-α gene expression in eWAT and circulating TNF-α in dietary-induced obese mice. Further, Ang 1–7 reduced TNF-α secretion in the lipotoxic model of macrophages. These results suggest that Ang 1–7 directly acts on adipose tissue macrophages to reduce TNF-α secretion. Macrophage-derived TNF-α not only impairs insulin signalling but also stimulates adipocytes to enhance MCP-1 secretion, indicating that the interaction between adipocytes and recruited macrophages forms a vicious cycle to augment inflammation [34,35]. Our results suggest that Ang 1–7 can break this vicious cycle by dual effect on adipocytes and macrophages (Figure 4b).

In summary, this study demonstrated that (1) Ang 1–7 improves insulin resistance and glucose tolerance in dietary-induced obese mice, (2) Ang 1–7 reduces MCP-1 at local (eWAT) and systemic levels, accompanied by a reduction in the number of CLS in eWAT of dietary-induced obese mice, (3) Ang 1–7 attenuates MCP-1 secretion in hypertrophic and TNF-α-exposed 3T3-L1 adipocytes, (4) Ang 1–7 reduces TNF-α at local (eWAT) and systemic levels in dietary-induced obese mice, and (5) Ang 1–7 attenuates TNF-α secretion in lipotoxic model of macrophages. Clinical trials on Ang 1–7 are underway for various diseases, including COVID-19 and cancer, and these trials have reported that Ang 1–7 is well tolerated and does not cause severe side effects [36,37]. Thus, the clinical translocation of Ang 1–7 in the context of obesity is feasible. Our findings further support the therapeutic potential of Ang 1–7 for addressing obesity and related metabolic disorders.

## 4. Materials and methods

### 4.1. Mice and treatments

The dietary-induced obese mouse model was created as described previously [38,39]. Four-week-old healthy male C57BL/6 mice (body weight, 15–18 g), purchased from CLEA Japan, were used for establishing the animal model. Mice were maintained at 24°C and a 12 h light/dark cycle with free access to food and water unless indicated. The animals were housed in cages with four mice in each cage and allowed to acclimatize for at least 5 days prior to starting the experiment. Mice were and fed an NC (CLEA Rodent Diet CE-2; CLEA Japan) or HFD (60 kcal% fat; Research Diets, Cat. No. D12492) for 8 weeks. Food intake was measured at the cage level, and the reported values represent the average intake per mouse, calculated by dividing the total food intake per cage by the number of mice in the cage. Ang 1–7 was purchased from Sigma-Aldrich (Cat. No. A9202) and administered to HFD-fed mice at a dose of 0.5 mg kg^−1^ body weight day^−1^ during the last 4 weeks of the study period via Alzet micro-osmotic pumps (model 1004, Cat. No. 0009922) implanted subcutaneously at the dorsum of the neck, as previously described [7]. Mice were randomly divided into three groups: NC group (mice fed NC), HF group (mice fed HFD), and HFA group (mice fed HFD and administered Ang 1–7). NC-fed and HFD-fed mice that did not receive Ang 1–7 underwent sham surgery. At 12 weeks, mice were randomly selected from each group and sacrificed to collect eWAT and blood. Mice were anesthetized through isoflurane (Pfizer) inhalation, following which blood was obtained via cardiopuncture. Plasma was separated via centrifugation at 4°C and stored at − 80°C until future analysis of TNF-α and MCP-1 (see below). eWAT was dissected and weighed and then frozen in liquid nitrogen and stored at − 80°C until further analysis. All animal experiments were performed in accordance with the Japanese Act on Welfare and Management of Animals. Our animal use protocol was approved by the Institutional Animal Care and Use Committee of Kyoto Prefectural University of Medicine (approval no. M2023–519). We adhere to the ARRIVE guidelines.

### 4.2. Glucose and insulin tolerance test

At 11 weeks of age, IPGTT and IPITT were performed as described previously [38]. For IPGTT, mice were injected with glucose (1 g/kg; Otsuka, Cat. No. 035079419) intraperitoneally after overnight fasting (18:00 to 10:00). For IPITT, mice were injected with insulin (0.75 U/kg; Eli Lilly, Cat. No. 428021922) intraperitoneally after 3 h of fasting (9:00 to 12:00). Blood was collected at 0, 30, 60, and 120 min for IPGTT and at 0, 15, 30, 60, 90, and 120 min for IPITT. Blood glucose was measured with a glucose compact analyser (Glutest Neo Alpha; Sanwa Kagaku Kenkyusho, Cat. No. 086532976).

### 4.3. Immunohistochemistry and quantification of CLS

Immunohistochemistry was performed as described previously [40]. Formalin-fixed, paraffin-embedded eWAT sections were incubated with F4/80 antibody (Cat. No. CL8940AP, 1:1000; Cedarlane Laboratories) for 60 min at 25°C, followed by incubation with biotinylated secondary antibody (Cat. No. BA-4000; Vector Laboratories). Secondary antibodies were visualized using the VECTASTAIN Elite ABC Standard Kit (Cat. No. PK-6100; Vector Laboratories). Slides were examined, and photomicrographs were captured under the same exposure and magnification using the all-in-one fluorescence microscope BZ-X710 (Keyence). CLS were identified as adipocytes completely surrounded by F4/80+ cells and were quantified in two randomly selected 40× fields per mouse.

### 4.4. Cell culture and treatment

The 3T3-L1 cell line (JCRB9014) was procured from the JCRB Cell Bank and cultivated in Dulbecco’s Modified Eagle’s Medium (DMEM, Gibco; Thermo Fisher, Cat. Nos.10565018 and 11885084) supplemented with 10% FBS (Gibco; Thermo Fisher, Cat. No. A5256701) and 100 U/mL penicillin plus 100 µg/mL streptomycin (Nacalai Tesque, Cat. No. 26253–84). Cells were cultured at 37°C in a 5% CO_2_ atmosphere. Adipogenesis was induced by cultivation in a culture medium containing 1 mM insulin (Nacalai Tesque, Cat. No. 12878–86), 0.5 mM IBMX (Nacalai Tesque, Cat. No. 19624–44), and 1.0 mM dexamethasone (Nacalai Tesque, Cat. No. 11107–64) for 2 days. Medium containing 1 mM insulin was replaced every 2 days for 6 days in total, after which the medium was changed to DMEM/10% FBS. 3T3-L1 adipocytes cultured for 1 and 3 weeks after differentiation have been previously utilized as non-hypertrophied adipocytes and hypertrophied adipocytes, respectively [16]. In the present study, 3T3-L1 adipocytes cultured for 1, 2, and 3 weeks after differentiation were treated with distilled water (DW), 10^−6^ mol/L Ang 1–7, or 10^−6^ mol/L Ang 1–7 and 10^−5^ mol/L A779 (Mas receptor antagonist; TCI AMERICA, Cat. No. A3281) for 24 h. The concentration of Ang 1–7 administration was determined according to previously reported methods [41]. Additionally, day 8 differentiated 3T3-L1 adipocytes exposed to TNF-α (Peprotech, Cat. No. 300-01a) for 24 h have been used as an *in vitro* model of insulin resistance [17]. TNF-α-exposed 3T3-L1 adipocytes were treated with DW, 10^−5^ mol/L Ang 1–7, or 10^−5^ mol/L Ang 1–7 and 10^−4^ mol/L A779 for 24 h in this study. The culture medium was subjected to enzyme-linked immunosorbent assay (ELISA) for analysing MCP-1.

Mouse monocyte/macrophage RAW264.7 cells (KAC Co. Ltd.) were seeded in 12-well plates and stabilized in DMEM supplemented with 10% FBS, 100 U/mL penicillin, and 100 µg/mL streptomycin. RAW246.7 cells were cultured in a humid atmosphere of 5% CO_2_ at 37°C. They were then treated with LPS (25 ng/mL, Sigma-Aldrich, Cat. No. L4391) and PA (100 µM, Wako, Cat. No. 165–00102) and utilized as a lipotoxic macrophage model, as previously described [42–44], and subsequently treated with DW (control) or 10^−6^ mol/L Ang 1–7 with or without 10^−5^ mmol/L A779 for 24 h. The culture medium was subjected to ELISA for assessing TNF-α secretion. In all the *in vitro* experiments, A779 was added 30 min before administrating Ang 1–7.

### 4.5. mRNA expression

To analyse mRNA expression, total RNA was isolated from eWAT using a NucleoSpin RNA II kit (Macherey-Nagel, Cat. No. 740955.50). Template cDNA was synthesized from 1 µg of total RNA with random hexamer primers as the template for each reaction in a ReverTra Ace qPCR RT Master Mix (Toyobo, Cat. No. FSQ201). Quantitative real-time PCR was performed as previously described [38]. The expression of target genes was examined by quantitative real-time PCR using TB Green Premix Ex Taq II (Tli RNaseH Plus; Takara, Cat. No. RR820A). An AB 7500 Real-Time PCR System (Applied Biosystems) was used for detecting fluorescence. Primer sequences are shown as follows: β-actin, forward primer: 5′-ATGCTCCCCGGGCTGTAT-3′, reverse primer: 5′-CATAGGAGTCCTTCTGACCCATTC-3′; MCP-1, forward primer: 5′-TCAGCCAGATGCAGTTAACGC-3′, reverse primer: 5′-TGATCCTCTTGTAGCTCTCCAGC-3′; TNF-α, forward primer: 5′- GGCAGGTCTACTTTGGAGTCATTGC-3′, reverse primer: 5′-ACATTCGAGGCTCCAGTGAATTCGG-3′; IL-6, forward primer: 5′-CCACTGCCTTCCCTACTTCA-3′, reverse primer: 5′-ACAGTGCATCATCGCTGTTC-3′; IL-1β, forward primer: 5′-TCTCACAGCAGCATCTCGAC-3′, reverse primer: 5′-CATCATCCCACGAGTCACAG-3′. β-actin was chosen as an internal standard.

### 4.6. ELISA

Mouse plasma insulin levels were measured using an ELISA kit (Morinaga Institute of Biological Science, Cat. No. M1104). Supernatants of 3T3-L1 adipocytes and RAW264.7 cells were obtained from cell-conditioned medium to measure TNF-α (R&D system, Cat. No. MTA00B) and MCP-1 (R&D system, Cat. No. MJE00B) by ELISA kits. All assays were performed according to the manufacturer’s instructions.

### 4.7. Statistical analysis

Statistical analyses were performed using the GraphPad Prism 7 software (GraphPad Software). Multiple groups were compared using repeated measurements of analysis of variance (ANOVA) with Tukey – Kramer post hoc test. Results are presented as mean ± SEM. Statistical significance was set at *p* < 0.05 and *p* < 0.01.

## Data Availability

The data presented in the manuscript are available at https://doi.org/10.6084/m9.figshare.27977577
